# Targeting Molecular Mechanism of Vascular Smooth Muscle Senescence Induced by Angiotensin II, A Potential Therapy via Senolytics and Senomorphics

**DOI:** 10.3390/ijms21186579

**Published:** 2020-09-09

**Authors:** Keisuke Okuno, Stephanie Cicalese, Katherine J. Elliott, Tatsuo Kawai, Tomoki Hashimoto, Satoru Eguchi

**Affiliations:** 1 Cardiovascular Research Center, Lewis Katz School of Medicine at Temple University, 3500 N. Broad Street, Philadelphia, PA 19140, USA; tun20059@temple.edu (K.O.); tug51315@temple.edu (S.C.); kelliott@temple.edu (K.J.E.); tuf88636@temple.edu (T.K.); 2 Department of Neurosurgery and Neurobiology, Barrow Aneurysm and AVM Research Center, Barrow Neurological Institute, Phoenix, AZ 85013, USA; Tomoki.Hashimoto@Barrowneuro.org

**Keywords:** vascular smooth muscle cells, angiotensin II, senolytic, aging

## Abstract

Cardiovascular disease (CVD) is a prevalent issue in the global aging population. Premature vascular aging such as elevated arterial stiffness appears to be a major risk factor for CVD. Vascular smooth muscle cells (VSMCs) are one of the essential parts of arterial pathology and prone to stress-induced senescence. The pervasiveness of senescent VSMCs in the vasculature increases with age and can be further expedited by various stressing events such as oxidative stress, mitochondria dysfunction, endoplasmic reticulum stress, and chronic inflammation. Angiotensin II (AngII) can induce many of these responses in VSMCs and is thus considered a key regulator of VSMC senescence associated with CVD. Understanding the precise mechanisms and consequences of senescent cell accumulation may uncover a new generation of therapies including senolytic and senomorphic compounds against CVD. Accordingly, in this review article, we discuss potential molecular mechanisms of VSMC senescence such as those induced by AngII and the therapeutic manipulations of senescence to control age-related CVD and associated conditions such as by senolytic.

## 1. Introduction

A discrepancy between lifespan and healthspan is becoming a global challenge as life-expectancy increases [1]. Aging is a major risk factor for cardiovascular diseases (CVD) such as hypertension, coronary and cerebral artery diseases [2]. The vascular system is damaged with age, demonstrating several signatures of vascular disorders [3]. Accumulating evidence support that cellular senescence contributes to the progression of cardiovascular pathologies along with age-related disorders [4]. It is important to note that the concept of early vascular aging has been developed to identify an individual with a high CVD risk who has a dissociation between chronological and biological vascular aging [5]. As a major hormone of the renin angiotensin system (RAS), angiotensin II (AngII) has been implicated in induction of premature cellular senescence in vessel wall, thus promoting early vascular aging [6,7].

Cellular senescence was first defined in the 1960s, where normal human fibroblasts lost the ability to replicate in culture at certain passages, indicating that cell senescence may be related to aging in vivo [8]. This type of senescence, termed replicative cell senescence, is a consequence of the critical loss of telomere length that occurs once somatic cells have undergone a maximal number of cellular division cycles [9]. Replicative senescence is associated with a persistent DNA damage response (DDR) [10] and induces cell senescence through p53/p21 and p16 ^INK4a^/the retinoblastoma protein (Rb), two parallel tumor suppressor signaling pathways [11].

Premature cellular senescence, termed stress-induced premature senescence (SIPS), can be achieved with non-chronological stress conditions [12]. Without detectable DDR, SIPS can be induced by various type of stressors including oxidative stress and metabolic stress [10]. Regardless of the induction mechanisms, senescent cells have some common (but not exclusive) features, which include permanent growth arrest, increased cell size, induction of a senescence-associated β-galactosidase (SA-βgal) activity, expression of a cyclin-dependent kinase inhibitor p16^INK4a^, formation of senescence-associated heterochromatin foci (SAHF), and senescence-associated secretory phenotype (SASP) [10]. Over time senescent cells accumulate from damage within tissue, most significantly at etiological sites of multiple diseases throughout the lifespan. Specifically, this includes insults to the cardiovascular system such as, atherosclerosis, diabetes, and heart failure [13]. SASP transmits persistent sterile inflammation that is associated with age-related chronic diseases and frailty, thus providing a novel therapeutic opportunity [14,15].

In this review, we focus on potential signaling and consequences of senescence in vascular smooth muscle cells (VSMCs) in response to AngII. We also advocate that the senescence mechanism will provide novel therapeutic targets to maintain healthy aging and/or counteract against CVD development.

## 2. SASP Regulation and the Secretory Phenotype of Senescent Vascular Cells

It is hypothesized that chronic SASP is the major contributor to aging associated organ dysfunction induced by senescent cells [14,15]. Cytokines, matrix remodeling enzymes, and extracellular vesicles have all been identified in senescent cell secretions, however the exact profile can change depending on the stressor [14,16,17]. Nuclear factor-κ B (NF-κB) and CCAAT/enhancer-binding protein (C/EBP-β) are classical mediators of SASP. Thus far varieties of SASP mediators have been reported which include toll-like receptors, an autocrine feed forward secretion of interleukin-1α (IL-1α), and epigenetic mediators such as high mobility group box 2 (HMGB2) and bromodomain-containing protein 4 (BRD4). In addition, the cellular nutrient sensing pathways, mammalian target of rapamycin (mTOR) and nicotinamide adenine dinucleotide (NAD^+^) are involved in SASP regulation (reviewed in [18]). Regarding SASP in vascular cell types, a prior study has demonstrated that senescent human aortic VSMCs secrete IL-1α and promote adjacent cells to a pro-adhesive and-inflammatory state in an autocrine manner [19]. However, in this study the findings are limited to replicative senescence and only one kind of stress-induced senescence (bleomycin) [19]. In another study using human coronary VSMCs, bleomycin-induced or replicative senescent cells were characterized including total cell lysates proteomics and qPCR, which demonstrated up-regulation of IL-1β, IL-6 and high mobility group box-1 (HMGB1). This study, however, did not confirm these factors to be secreted upon senescence [20]. In addition, arterial VSMCs isolated from children with chronic kidney disease showed elevated senescence and SASP, which include IL-6 and calcification promoting bone morphogenetic protein-2 and osteoprotegerin [21]. It is also important to note the paracrine mechanisms of SASP potentially occur in VSMCs via other senescent cell types such as endothelial cells [22], fibroblasts, immune cells and adipocytes as these are all cellular communicators of the vasculature. These recently recognized cell communications are considered as key parts of inflamm-aging; a chronic low-grade inflammation associated with aging and aging-related diseases [23]. Interestingly extracellular vesicles also termed exosome has been recently implicated as a part of SASP in some cell systems [16,24] including endothelial cells [25]. In particular, functional as well as proteomic characterization of exosomes have been performed and compared in aortic VSMCs and endothelial cells. Endothelial cell-derived exosome but not VSMC-derived exosome caused HMGB1-dependent inflammatory responses and senescence in VSMCs [26] suggesting a paracrine role of vascular exosomes in mediating senescence and SASP. Regarding the AngII-induced senescence, removal of senescent cells by a senolytic ABT737 attenuated AngII-induced leukocyte adhesion in cultured endothelial cells suggesting a contribution of SASP in endothelial dysfunction induced by AngII [27]. As only a limited percentage of endothelial cells undergo senescence upon AngII exposure, these findings suggest potential paracrine role of SASP in AngII-induced cardiovascular pathology [27]. However, further research is needed to characterize vascular cell SASP and its consequences with more pathologically relevant conditions.

## 3. Seno-modulation against Cardiovascular Aging

Our understanding of the molecular mechanism leading to senescence is becoming much clearer, particularly those in CVD [7,28,29]. Moreover, genetic or pharmacological removal (senolytics) or prevention (senomorphics) appears to be a powerful tool to investigate contribution of senescence in CVD [29]. In addition, drugs to attenuate the SASP network have been proposed as senostatics, which may also be a therapeutic against age-related diseases [30]. Genetic removal of senescent cells in mice have been developed such as p16^Ink4a^-ATTAC mice in which selective apoptosis can be induced in senescent cells demonstrating improvements in aging related disorders [31] and extension of healthy lifespan [32]. Similarly, p16-3MR mice with low density lipoprotein receptor (Ldlr) −/− background as well as p16^Ink4a^-ATTAC Ldlr−/− mice, removal of senescent cells prevented atherosclerosis development [33]. Pharmacological removal of senescent cells by ABT263 which inhibits anti-apoptotic proteins Bcl-2 and Bcl-x and selectively kills senescent cells [34] was also effective against atherosclerosis in Ldlr−/− mice [33]. In addition, endothelial p53 deletion prevented cardiac fibrosis and heart failure in response to pressure overload in mice [35] and protected systemic metabolic disorders in mice fed with high caloric diet [36]. Systemic removal of senescent cells in aged mice by ABT263 protected mice from cardiac hypertrophy and heart failure upon experimental myocardial infraction [37]. It is important to note that immune system has an endogenous defense mechanism to remove excess senescent cells via immunosurveillance, which leads to a development of immunotherapies to eliminate senescent cells [38]. The recent most exciting advancement in this field may be the development of engineered T cells expressing a chimeric antigen receptor (CAR T cells) to target senescence in vivo. The urokinase-type plasminogen activator receptor (uPAR) was identified as a selective cell surface marker expressed in senescent cells [39]. CAR T cells targeting uPAR appears effective against senescent malignant cells in vivo as well as liver fibrosis in animal models of non-alcoholic steatohepatitis, a severe form of fatty liver disease [39]. Contrary to these finding suggesting detrimental roles of senescence, p53/p16^INK4a^ double knockout mice showed accelerated cardiac fibrosis upon pressure overload even while the induction of senescence was prevented [40]. Clinical trials utilizing senolytics are currently ongoing for several chronic diseases [41].

Although senolytic effects have been investigated in CVD models, limited information is available regarding the potential application of senomorphics or senostatistics in vascular cells or animal models of chronic diseases. A prior review article defined senomorphics as agents that should prevent either senescence induction or SASP without induction of senescent cell apoptosis [42]. According to this definition, effects of several candidate compounds of senomorphics on senescence, SASP and age-related diseases have been described [42]. However, none of these compounds were originally designed as senomorphics. Among the potential targets to control SASP by senostatics, cGMP-AMP synthase (cGAS), a cytosolic DNA sensor that activates innate immunity and stimulator of interferon genes (STING) signaling pathway has recently been identified as a critical SASP regulator [18]. Retrotransposons are a special class of parasitic genetic elements that can replicate their DNA within our genes. One of the successful retrotransposons is the class of long interspersed nuclear element 1 (Line1). These 6 kb, fully functional retrotransposons can replicate not only themselves and accumulates by age and aging-related diseases, but accumulation of Line1 cDNA triggers strong type I interferon response via activation of cGAS-STING leading to SASP [43]. This response can be antagonized by nucleoside reverse transcriptase inhibitors (NRTIs) that inhibit the L1 reverse transcriptase. The NRTI lamivudine was able to attenuate SASP in senescent cells and age-associated inflammation in mice [43,44]. Thus, NRTI could be a promising candidate for senostatics. At this point, the effects of the senomorphics and or senostatics on CVD remain unclear. Further research on the potential application and mechanistic investigation with seno-modulatory interventions is strongly desired.

## 4. Classical vs. Novel RAS in Aging

**Figure 1:**
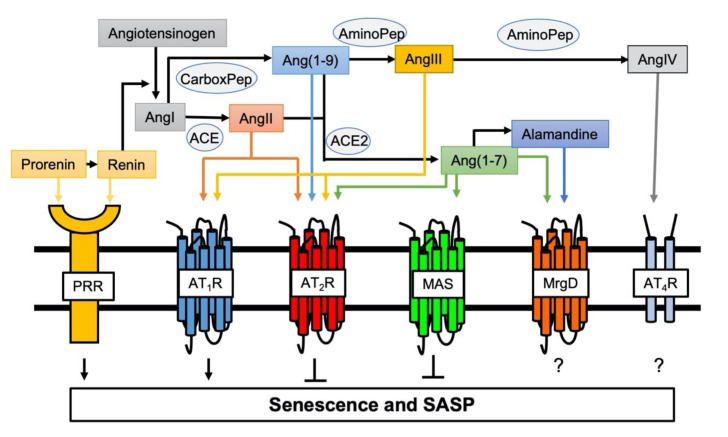
Involvement of novel RAS peptides and receptors in senescence regulation. Classical RAS via activation of AT_1_R positively regulates induction of senescence [55,56]. In contrast, AT_2_R protects against AT_1_R-mediated senescence [50]. Pro (renin) receptor (PRR) is a new member of the RAS receptors and mediates senescence [51]. ACE2 cleaves AngII and Ang(1–9) to produce Ang(1-7) to initiate protective arm of novel RAS (the ACE2/Ang(1-7)/MAS axis), which counter-regulates AT_1_R-mediated senescence [52,53]. The roles of additional Ang(1-7) receptor MrgD and AngIV receptor (AT_4_R) play in senescence remain unknown.

General mechanisms contributing to arterial aging include mitochondrial dysfunction, oxidative stress, inflammation, and activation of the RAS [45]. Chronic RAS activation leads to damage in several organs, which is associated with aging and oxidative stress in cells or mitochondria [46]. Aging also causes enhancement in the activity of and response to the RAS. [47]. AngII is the essential hormone acting through two receptor subtypes, AngII type 1 receptor (AT_1_R) and AngII type 2 receptor (AT_2_R). Activation of AT_1_R promotes the majority of RAS physiology and pathophysiology including vasoconstriction, cardiovascular hypertrophy and fibrosis, inflammation and oxidative stress [48]. While there are some exceptions and the exact signaling mechanisms are still unclear, activation of AT_2_R generally counteracts the classical RAS actions including cell growth, oxidative stress, inflammation and fibrosis [48,49]. Moreover, enhancement of senescence markers induced by AngII was observed in VSMCs cultured from AT_2_R deficient mice suggesting the anti-senescence role for AT_2_R [50]. In addition, for the past two decades, significant advancement has been made in understanding the function and mechanisms utilized by new members of the angiotensin family ligands and receptors [48]. Among them, using systemic pro (renin) receptor transgenic mice, it was demonstrated that pro (renin) receptor mediates skeletal muscle atrophy and the feature of sarcopenia by inducing muscle senescence via Wnt/β-catenin signal activation [51]. In contrast, the angiotensin converting enzyme 2 (ACE2)/Ang(1-7)/Mas receptor arm of RAS generally counter-regulate the classical RAS function (Figure 1) [48]. ACE2 deficiency in mice accelerated age-related muscle weakness and the associated senescence phenotype as a model of sarcopenia, whereas Ang(1-7) treatment restored this [52,53]. Protection against AngII- as well as IL-1β-induced senescence by Ang(1-7)-dependent activation of Mas receptor was also shown in endothelial cells [54].

Previous reports have demonstrated that expression of AT_1_R increases with age, whereas AT_2_R decreases with age in rodents [47,57]. As lifespan can be extended with AT_1_R (AT_1a_R) knockout as well as treatment with AT_1_R blockers in rodents [58,59], it is reasonable to speculate the critical role AT_1_R has in mediating cardiovascular aging. The mechanism of the lifespan extension seems to involve preservation of mitochondrial numbers [58,59]. Accordingly, inhibition of the classical RAS by an ACE inhibitor appears effective in attenuating frailty and systemic inflammatory responses in aged mice [60]. Analyses on ACE polymorphism [61] and AT_1_R polymorphism [62] further support the critical negative relationship between AngII/AT_1_R and healthspan in humans. The AngII antagonists are the most frequently used anti-hypertensives. However, only circumstantial information is available regarding the anti-aging actions of AngII antagonists in humans [63,64]. While it is challenging, more translational research is encouraged on the RAS system in humans, as there are new opportunities to design and evaluate the efficacies of these drugs against human aging or premature aging populations [65].

## 5. Signaling Mechanism of Senescence Induced by AngII in Cultured VSMC

**Figure 2:**
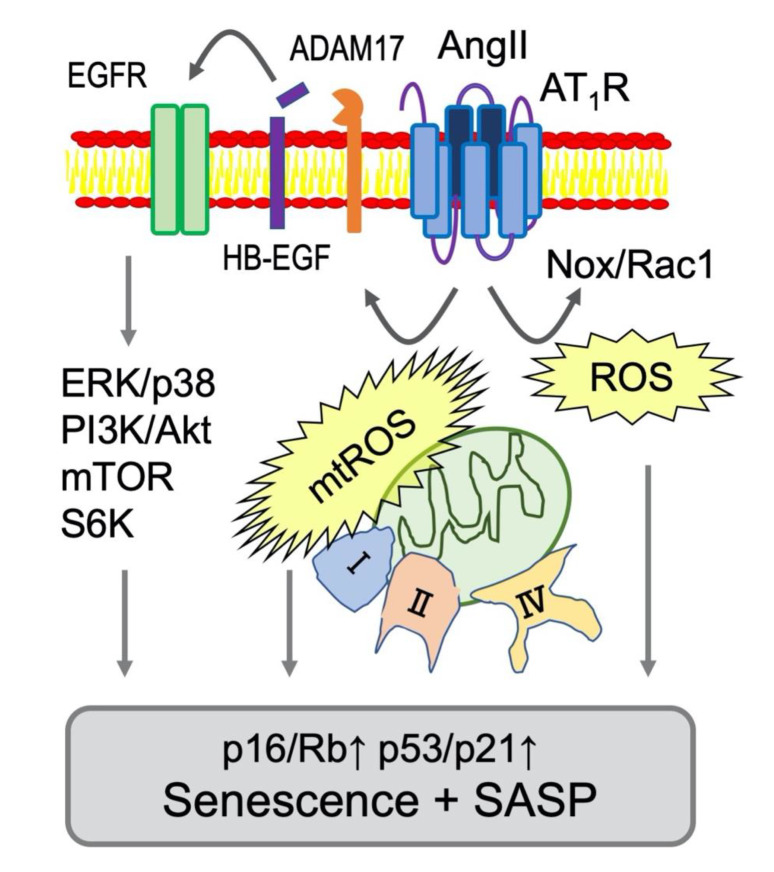
Signaling mediators of AngII-dependent senescence in VSMCs. Nox-derived ROS production is activated by AngII through AT_1_R and a small GTPase Rac1 [66,67]. In VSMCs, mitochondrial complex I or II were stimulated by AngII causing production of mitochondrial ROS (mtROS) and subsequent senescent responses [68]. In addition, mitochondrial respiratory disfunction occurred at complex IX was reported to induce senescence via p53 [69]. Moreover. Major Ser/Thr kinases involved in senescence (extracellular signal-regulated kinase (ERK), p38, mTOR and S6K) are activated via ADAM17-dependent EGFR transactivation in VSMCs [48]. These signaling events are considered major drivers of AT_1_R mediated vascular senescence and SASP (Table 1 for the references).

**Figure 3:**
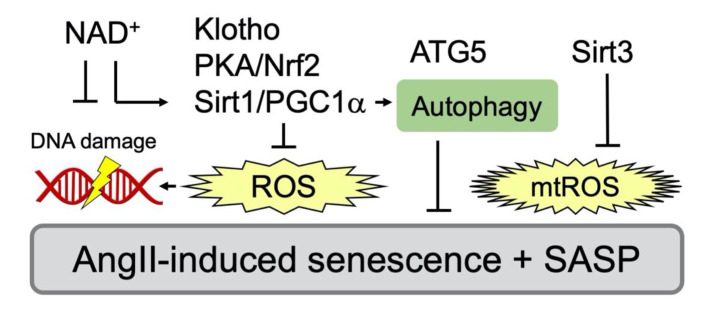
Inhibitory signaling events against AngII/AT_1_R-mediated senescence in VSMC. Various inhibitors that attenuate senescence via antagonizing oxidative stress have been reported. PGC-1α deficiency promotes vascular senescence with increased ROS and results in reduced expression of Sirt1 [76]. PGC-1α also maintains autophagy and a component of autophagy, autophagy related 5 (ATG5) to counter-regulate senescence [77]. Nrf2 plays a key role in protection of cellular senescence via PKA/CREB pathway [78]. Soluble Klotho, an antiaging hormone, attenuates AngII-induced VSMC senescence through Nrf2 induction and ROS attenuation [79]. Mitochondrial Sirt3 also protects against mtROS [76,80]. In addition, reduction in NAD^+^ exaggerates AngII-induced vascular senescence [76,81].

AngII-induced senescence of VSMCs appears to be mediated through several signaling mechanisms involving nicotinamide adenine dinucleotide phosphate (NADPH) oxidase, reactive oxygen species (ROS) and mTOR (Figure 2 and Table 1). In contrast, activation of autophagy, cyclic AMP (cAMP)/protein kinase A (PKA), sirtuins, nuclear factor erythroid 2-related factor 2 (Nrf2) and klotho counter-regulate AngII-induced senescence via several distinct mechanisms (Figure 3 and Table 2). The following section will aid in explaining the inter-relationship of these signaling events and whether they are required (needed factors) or sufficient (main driver of the alteration) for the phenotypes.

### 5.1. Contribution of Oxidative Stress

The molecular insight of SIPS induced by AngII had been limited in demonstrating that AngII induced SIPS via AT_1_R and p21/p53 induction in VSMCs [55,56]. Oxidative stress in the vasculature is considered as a primary mechanism underlying vascular dysfunction and aging. Increases in arterial ROS leads to elevated expression and activity of nicotinamide adenine dinucleotide phosphate (NADPH) oxidase (Nox), as well as increased endothelial nitric oxide synthase (eNOS) uncoupling [85]. In VSMCs, Nox is activated by AngII via AT_1_R leading to superoxide production and subsequent activation of downstream tyrosine and serine/threonine kinases [86,87]. AngII also increases superoxide production from mitochondria by increasing activity of the electron transfer chain as well as Nox in vascular cells [88]. In VSMCs, pharmacological inhibition of mitochondrial complex I or complex II, or treatment with the mitochondrial ROS scavenger significantly reduced superoxide production as well as senescence assessed with SA-βgal staining in VSMCs [68]. However, Nox1 siRNA [70,71] as well as pharmacological inhibitors of serine/threonine kinases (Akt, mitogen-activated protein kinase kinase (MEK) and p38 mitogen-activated protein kinase (MAPK)) [72] also attenuated AngII-enhanced SA-β gal staining in VSMCs. A small GTPase Rac1 is a contributing component of Nox activation complex. Inhibition of AngII-induced VSMC senescence via Rac1 inhibitor NSC23766 has been interpreted for its ROS mitigation [73]. Nrf2 is a critical transcriptional factor, which mediates antioxidant defense programs upon oxidative stress via binding to antioxidant response elements. It can be also activated by cAMP/PKA responsible CREB binding protein. A glucagon-like peptide-1 analog appears to attenuate AngII-induced VSMC senescence via PKA-dependent Nrf2 activation [78]. Klotho gene delivery is known to suppress AngII-induced Nox2 expression in VSMCs [89]. Soluble Klotho further attenuates AngII-induced VSMC senescence via Nrf2 induction [79]. In addition, AngII-induced VSMC senescence appears to require inhibition of Forkhead box (Fox)O1 transcriptional activity to induce silence information regulator 2-like 1 (Sirt1) via peroxisome proliferator-activated receptor gamma coactivator (PGC)-1α (PGC1-α) serine phosphorylation. This Sirt1 inhibition reduces anti-oxidative catalase expression leading to VSMC senescence [76]. In VSMCs, the activation of α7 nicotinic acetylchoine receptor activation is shown to attenuate AngII-induced VSMC senescence by NAD^+^-dependent Sirt1 activation [82]. Taken together, these data support the overall roles of Nox and ROS in AngII-induced senescence in VSMCs. Nox and ROS in AngII signaling and CVDs have been studied more than 25 years, albeit not in relation to senescence [66,67]. It seems that the Nox/ROS dependent mechanism of senescence could be only a minor portion of the ROS/Nox functions in the AngII signaling system and they may be required but not sufficient for VSMC senescence induced by AngII.

### 5.2. mTOR and Autophagy

mTOR is a master coordinator of cell growth and metabolic adaptation. mTOR is a serine/threonine protein kinase that consists of the catalytic subunit of two distinct protein complexes, that are mTOR complex 1 (mTORC1) and 2 (mTORC2) [90]. Several lines of evidence support the direct relationship between mTORC1 and senescence. Reduction in mRNA translation during mTORC1 inhibition slows senescence by suppressing the accumulation of proteotoxic and oxidative stress. Inhibition of mTORC1 also slows senescence by increasing autophagy, which helps clear damaged proteins and organelles of mitochondria [91]. Accordingly, contribution of mTOR and protective autophagy to AngII-induced VSMC senescence has been demonstrated by using mTOR inhibitor, rapamycin, and autophagy inhibitors, 3-methyladenine and bafilomycin A1 [83]. The phosphatidylinositol-3-kinase (PI3K)/Akt pathway positively regulates mTOR activity. AngII-induced VSMC senescence appears to require signal communication between PI3K/Akt and an actin binding protein, smooth muscle 22α (SM22α). This signal communication leads to Akt-dependent phosphorylation and inhibition of mouse double minute 2 homolog (Mdm2) which mediates ubiquitination and degradation of p53 [74]. In addition, PGC1-α appears necessary to maintain autophagy in VSMCs, where AngII-induced senescence was enhanced by another autophagy inhibitor, spautin-1, or by siRNA silencing of autophagy components autophagy related 5 (ATG5) or p62/sequestosome 1 [77]. However, conflicting findings have been published regarding whether AngII positively or negatively regulates autophagy in VSMCs and whether the VSMC autophagy is protective or detrimental for AngII-regulated CVDs [49,92].

Outside of senescence, AngII-induced mTOR activation has been implicated in protein synthesis in VSMCs via S6 kinase activation [93,94]. Ribosomal S6 kinase encoded by *S6KI* is one of the major effectors of mTOR1. In VSMCs, the PI3K/Akt/mTOR/S6K cascade activation via AT_1_R is primarily mediated by the epidermal growth factor receptor (EGFR) transactivation [48]. Interestingly, *S6KI* deficient mice is protected against age or diet-induced obesity while enhancing insulin sensitivity [95], have extended life span and recapitulate the phenotype seen with caloric restriction or pharmacological AMPK activation [96]. A nuclear epigenetic factor ZRF1 has been recently identified as a novel substrate for S6 kinase, and mediates the S6K-dependent senescence program [97]. These findings thus deserve further investigation for the relationship of the S6K cascade to VSMC senescence.

### 5.3. Vascular Senescence in Vivo by AngII Infusion

AngII infusion has been shown to induce SA-βgal in aortas of hyperlipidemic mouse models such as apolipoprotein E (ApoE) deficient mice [55]. AngII also caused vascular senescence in a collagen I mutant mouse in which the vascular senescence correlated with aortic stiffness [98]. Similar to findings in a cell culture model, VSMC Sirt1 appears protective against vascular senescence during abdominal aortic aneurysm (AAA) development following AngII infusion in ApoE deficient mice [84]. In addition, the aortic senescence induced by AngII infusion in Ldlr deficient mice was attenuated by concurrent sm22α silencing because sm22α interacted with and inhibited MDM2 which control ubiqutination and degradation of p53 [74]. The role for p53 in AAA development and aortic senescence was also confirmed with p53 deficient mice as well as caloric restriction [69]. Mitochondrial Sirt3 protects against oxidative stress by promoting deacetylation of mitochondrial superoxide dismutase, SOD2. Importance of mitochondrial-derived ROS in vascular oxidative stress and senescence has been confirmed with mitochondrial Sirt3 deficient mice and Sirt3 transgenic mice infused with AngII [80]. Generation of NAD^+^ via nicotinamide phosphoribosyl transferase (Nampt) is known to mediate cell vitality. In mice deficient with smooth muscle Nampt, AngII induced aortic wall degeneration and dissection was exaggerated, which was associated with senescence [81]. Taken together, these studies identify the link between AngII and VSMC senescence in vivo, however it is desired to further test pharmacological or genetic senolytic (or senomorphic) treatment in AngII-dependent CVD models such as hypertension and AAA.

### 5.4. Mitochondrial Dynamics, Endoplasmic Reticulum Stress and Senescence

Mitochondrial dysfunction including mitochondrial oxidative stress is a hallmark of the aging process, however, recent evidence has emerged that dysfunctional mitochondrial quality control (fission/fusion, biogenesis and mitophagy) also contribute to senescence [99,100]. A small GTPase, dynamin-related protein 1 (Drp1) is one such molecule that mediates mitochondrial fission, and excessive Drp1 activity and mitochondrial fission are associated with several models of CVD [101]. Recently, involvement of Drp1 in senescence induction was demonstrated [102,103]. In abdominal aortic smooth muscle cells, AngII stimulated Drp1 activity and mitochondrial fission via ERK1/2-dependent Drp1 Ser616 phosphorylation. Inhibiting Drp1 activity in mice further attenuated AngII-induced AAA development which was associated with reduction of aortic senescence assessed by SA-βgal and p16 induction [75]. In endothelial cells, Drp1 also mediates NF-κB activation [104]. In addition, mitochondrial fission often associates with the turnover of damaged mitochondria through mitophagy, however, the relationship between mitophagy and senescence remains to be explored. Interestingly, the concept of a subtype of senescence has been recently proposed as MiDAS (mitochondrial dysfunction-associated senescence) which demonstrates an altered SASP proteome [105]. Therefore, understanding the potential roles of MiDAS SASP elicited by AngII over conventional SASP in cardiovascular dysfunction may also be beneficial.

**Figure 4:**
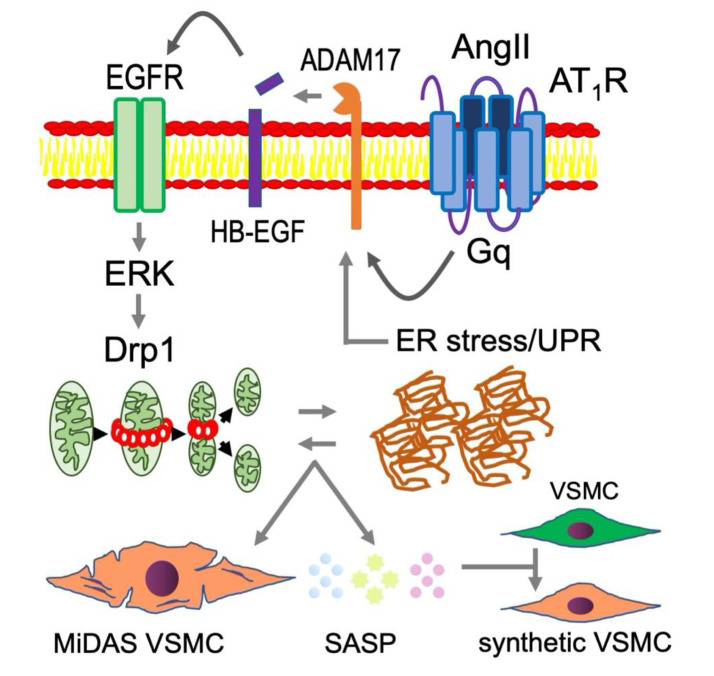
Mitochondrial fission and ER stress likely play upstream roles in AngII-induced vascular senescence and SASP contributing to RAS-mediated pathophysiology in CVDs. In addition to the molecular mechanisms illustrated in Figure 2, mitochondrial fission and ER stress are enhanced under chronic RAS activation contributing to VSMC senescence [6]. It is hypothesized that MiDAS VSMC produce altered SASP, which adversely affects non-senescent VSMC phenotype leading to enhancement of vascular remodeling. Such VSMC phenotype has been described as “smooth muscle cell- or arterial- stiffness syndrome” [112,113].

The endoplasmic reticulum (ER) maintains cellular proteostasis, and is another organelle that is highly involved in aging of the cell. Evidence exists that ER stress and subsequent unfolded protein response (UPR) mediates induction of major senescence markers including SA-βgal. Moreover, because the ER is responsible for the trafficking and secretion of many proteins, ER stress alters the SASP composition while also propagating proteotoxicity and UPR signaling to dictate SASP secretions [106]. In terms of vascular senescent cell development, investigations of the link between mitochondrial and ER are only just beginning. In cultured endothelial cells AngII/AT_1_R-induced senescence appears to involve interaction between Drp1 and ER stress [27]. AngII-induced ER stress causes protein aggregation in VSMCs [107]. Furthermore, the lamin A variant progerin accelerates aging, and has been shown to induce ER stress and UPR activation in aortic VSMCs, which overall exacerbates atherosclerosis [108]. Vascular ER stress is commonly associated with arterial stiffness [109]. AngII-induced EGFR transactivation mediates ER stress and subsequent vascular remodeling [110,111]. Accordingly, EGFR, mitochondrial fission/Drp1 and ER stress likely play upstream roles in AngII-induced vascular senescence and SASP thus contributing to RAS-mediated pathophysiology in CVDs (Figure 4).

## 6. Limitation and Future Direction

In vivo studies regarding the contribution of senescence to cardiovascular dysfunction and diseases are still limited in certain animal models including mice with AngII infusion. In these models, most interventions are not directed towards senescence specifically and show instead only the association between senescence and the phenotype alterations. In mice infused with AngII, aortic senescence appears limited whereas the condition is sufficient to induce cardiovascular pathology including hypertension, aortic stiffness, perivascular fibrosis and cardiac hypertrophy. This may mean that senescence is not an essential process for AngII-induced pathophysiology or more likely that the mice used may be too young for the experiments to test whether senescence has any relevance in the AngII pathology. Accordingly, development of better animal models which simulate human premature aging seems essential for testing the effectiveness of senolytics or senomorphics against CVD. It should be noted that in senescence accelerated mice, AngII plus CaCl_2_-induced AAA appear enhanced [114].

## 7. Conclusions

Aging is a nonmodifiable risk factor for CVD. Since the lifespan of our population increases, the urgency to study vascular aging as a therapeutic target is evident. Accumulation of senescent cells causes production of pathological inflammatory molecules via SASP and negatively affects vascular homeostasis. Some anti-senescence therapies suppress the induction of senescent cells not only in vitro but in vivo through various pathways as we discussed. We believe that adapting novel anti-senescent approach into the clinic has potential to effectively prevent or treat CVD. Changes in mitochondrial function and ER stress appear associated with senescence. Therefore, in addition to explore new classes of senolytics and senomorphics, future studies should investigate a novel anti-aging therapy that target multiple organismal phenotypes in senescence. Moreover, development of new animal model to simulate CVD linked to human aging in which we can test the effectiveness of anti-aging therapies are desired.

## Figures and Tables

**Table 1:** Mediators of AngII-dependent senescence in VSMC.

System	Inducer	Signaling	Reference
in vitro	Nox-derived ROS	Nox1/2	[70,71]
in vitro	Nox-derived ROS	MEK/ERK, p38, Akt	[72]
in vitro	Nox-derived ROS	Rac1	[73]
in vitro	Mitochondrial ROS	Complex I/II	[68]
in vitro	Sm22α	PI3K/Akt	[74]
in vivo	p53	Complex IV	[69]
in vivo	Mitochondria fission	Drp1	[75]

**Table 2:** Inhibitors of AngII-dependent senescence in VSMC.

System	Inhibitor	Target	Reference
in vitro	PGC1-α/Sirt1	Oxidative stress	[76]
in vitro	Sirt1	Oxidative stress	[82]
in vitro	PKA/Nrf2	Oxidative stress	[78]
in vitro	Klotho/Nrf2	Oxidative stress	[79]
in vitro	PGC1-α	Atg5/autophagy	[77]
in vitro	autophagy	Oxidative stress	[83]
in vivo	Sirt1	p21/AAA	[76,84]
in vivo	Sirt3	Mitochondrial ROS	[76,80]
in vivo	NAD^+^	DNA damage	[76,81]

## Abbreviations

AAA	Abdominal aortic aneurysm
ACE	Angiotensin converting enzyme
AngII	Angiotensin II
ApoE	Apolipoprotein E
ARDS	Acute respiratory distress syndrome
AT_1_R	Angiotensin II type 1 receptor
AT_2_R	Angiotensin II type 2 receptor
ATG5	Autophagy related 5
BRD4	Bromodomain-containing protein 4
CAR T cells	T cells expressing a chimeric antigen receptor
C/EBP-β	CCAAT/enhancer-binding protein
cGAS	cGMP-AMP synthase
COVID-19	Coronavirus disease 2019
CVD	Cardiovascular disease
DDR	DNA damage response
Drp1	Dynamin-related protein 1
EGFR	Epidermal growth factor receptor
eNOS	Endothelial nitric oxide synthase
ER	Endoplasmic reticulum
ERK	Extracellular signal-regulated kinase
HMGB	High mobility group box
IL-1α	Interleukin-1α
Ldlr	Low density lipoprotein receptor
Line1	Long interspersed nuclear element 1
MAPK	Mitogen-activated protein kinase
Mdm2	Mouse double minute 2 homolog
MEK	Mitogen-activated protein kinase kinase
MiDAS	Mitochondrial dysfunction-associated senescence
mTOR	Mammalian target of rapamycin
mTORC1	mTOR complex 1
NADPH	Nicotinamide adenine dinucleotide phosphate
Nampt	Nicotinamide phosphoribosyl transferase
NF-κB	Nuclear factor-κB
Nrf2	Nuclear factor erythroid 2-related factor 2
NRTI	Nucleoside reverse transcriptase inhibitor
Nox	Nicotinamide adenine dinucleotide phosphate (NADPH) oxidase
PGC1-α	Peroxisome proliferator-activated receptor gamma coactivator (PGC)-1α
PI3K	Phosphatidylinositol-3-kinase
PKA	Protein kinase A
RAS	Renin angiotensin system
Rb	Retinoblastoma protein
ROS	Reactive oxygen species
SA-βgal	Senescence-associated β-galactosidase
SAHF	Senescence-associated heterochromatin foci
SARS-CoV	Severe acute respiratory syndrome coronavirus
SASP	Senescence associated secretory phenotype
SIPS	Stress-induced premature senescence
Sirt1	Silence information regulator 2-like 1
SM22α	Smooth muscle 22α
SOD2	Mitochondrial superoxide dismutase
STING	Stimulator of interferon genes
uPAR	Urokinase-type plasminogen activator receptor
UPR	Unfolded protein response
VSMCs	Vascular smooth muscle cells
